# Heterogeneous natural selection on oxidative phosphorylation genes among fishes with extreme high and low aerobic performance

**DOI:** 10.1186/s12862-015-0453-7

**Published:** 2015-08-26

**Authors:** Feifei Zhang, Richard E. Broughton

**Affiliations:** Oklahoma Biological Survey and Department of Biology, University of Oklahoma, 111 E Chesapeake Street, Norman, OK 73019 USA

## Abstract

**Background:**

Oxidative phosphorylation (OXPHOS) is the primary source of ATP in eukaryotes and serves as a mechanistic link between variation in genotypes and energetic phenotypes. While several physiological and anatomical factors may lead to increased aerobic capacity, variation in OXPHOS proteins may influence OXPHOS efficiency and facilitate adaptation in organisms with varied energy demands. Although there is evidence that natural selection acts on OXPHOS genes, the focus has been on detection of directional (positive) selection on specific phylogenetic branches where traits that increase energetic demands appear to have evolved. We examined patterns of selection in a broader evolutionary context, i.e., on multiple lineages of fishes with extreme high and low aerobic performance.

**Results:**

We found that patterns of natural selection on mitochondrial OXPHOS genes are complex among fishes with different swimming performance. Positive selection is not consistently associated with high performance taxa and appears to be strongest on lineages containing low performance taxa. In contrast, within high performance lineages, purifying (negative) selection appears to predominate.

**Conclusions:**

We provide evidence that selection on OXPHOS varies in both form and intensity within and among lineages through evolutionary time. These results provide evidence for fluctuating selection on OXPHOS associated with divergence in aerobic performance. However, in contrast to previous studies, positive selection was strongest on low performance taxa suggesting that adaptation of OXPHOS involves many factors beyond enhancing ATP production in high performance taxa. The broader pattern indicates a complex interplay between organismal adaptations, ATP demand, and OXPHOS function.

**Electronic supplementary material:**

The online version of this article (doi:10.1186/s12862-015-0453-7) contains supplementary material, which is available to authorized users.

## Background

Physiological processes may serve as mechanistic links between genotypes and organismal phenotypes. Accordingly, adaptations in genes of energy metabolism pathways may facilitate the evolution of organismal structures and life habits with diverse energy requirements. Proteins encoded by the mitochondrial genome serve as core subunits of the oxidative phosphorylation (OXPHOS) system, the primary source of ATP in eukaryotic cells. Consequently, organismal traits with differing ATP demands may be influenced by adaptation in mitochondrial genomes [1–5]. Our understanding of patterns and rates of adaptive change in mitochondrial genomes remains limited despite extensive use of mitochondrial genes as molecular markers in evolutionary studies [6] and the important role of mitochondria in many human pathologies [7]. Although there is ample evidence of natural selection acting on mitochondrial genes [8–15], the functional significance of adaptive mitochondrial change is rarely known.

Evidence of positive selection on OXPHOS genes has been associated with evolution of a variety of energetically demanding characteristics (reviewed in [16]), including origin of large brains in anthropoid primates [17], powered flight in bats [18], and adaptation to cold environment in polar bears [19]. These suggest a significant role for OXPHOS in organismal adaptation, and because divergence among lineages often involves traits with different energy usage, OXPHOS evolution may be an important factor in the diversification of life.

OXPHOS functional efficiency may be particularly important to energy intensive processes such as locomotion. Variation in locomotive performance of fishes is among the most extreme among vertebrates, ranging from largely sedentary filter feeders and sit-and-wait predators to highly migratory species and active pelagic foragers. For example, seahorses and flounders spend much of their time nearly motionless, while tunas and marlins are “high-performance” swimmers that exhibit high aerobic metabolism and prolonged fast swimming [20, 21]. Highly-active fish taxa exhibit many morphological and physiological adaptations that enhance swimming performance (reviewed in [22]). Such adaptations include modifications of body shape and hydrodynamics [23–25], swimming form and mechanical kinematics [26–28], muscle composition [29–31], metabolic rates [32], heart volume and aerobic capacity [32–34], and mitochondrial structure and concentration [35]. However, much less is known about the molecular adaptations that influence organismal performance.

At the molecular level, expression levels of proteins directly involved in energy metabolism may be increased in highly mobile fish species. Tunas and marlins, which have higher cruising speeds than other active fishes [21], have been shown to have elevated myoglobin [36] and higher concentrations of metabolic enzymes in heart and skeletal muscle (reviewed in [36]). Elevated activities of citrate synthase (which catalyzes the first reaction of the Krebs cycle), carnitine-palmitoyl transferase, and 3-hydroxy-o-acyl-CoA dehydrogenase (rate-limiting enzymes in fatty acid oxidation), reflect the increased aerobic metabolic potential of scombrid fishes [35]. In addition, OXPHOS genes are differentially expressed between morphs of lake whitefish that differ in activity levels in foraging behavior [37].

Alternatively, or in addition, to variation in gene expression and post-transcriptional modification, divergent energy demands may lead to adaptive evolution in the structure of specific OXPHOS proteins. Variation in OXPHOS proteins could influence the efficiency of ATP production by affecting how tightly electron transport and proton pumping are coupled in the electron transport chain. Modifications in the structure of OXPHOS complexes I–IV caused by amino acid substitutions in constituent proteins could affect “slip reactions”, resulting in more or less protons pumped by the electron-transport-chain for each electron pair transferred (the H^+^/2e ratio) (reviewed in [38]). Alternatively, substitutions in proteins of ATP synthase could modify the amount of ATP made for each proton driven through it (the H^+^/ATP ratio) [38]. These phenomena are consistent with prior reports of positive selection in the cytochrome c oxidase genes of the high performance billfishes [39, 40], the *MT-ND2* and *MT-ND5* genes of some migratory Pacific salmon [41], and *MT-ND2*, *MT-ND4* and *MT-ND5* genes of pelagic Atlantic herring [42].

**Fig. 1:**
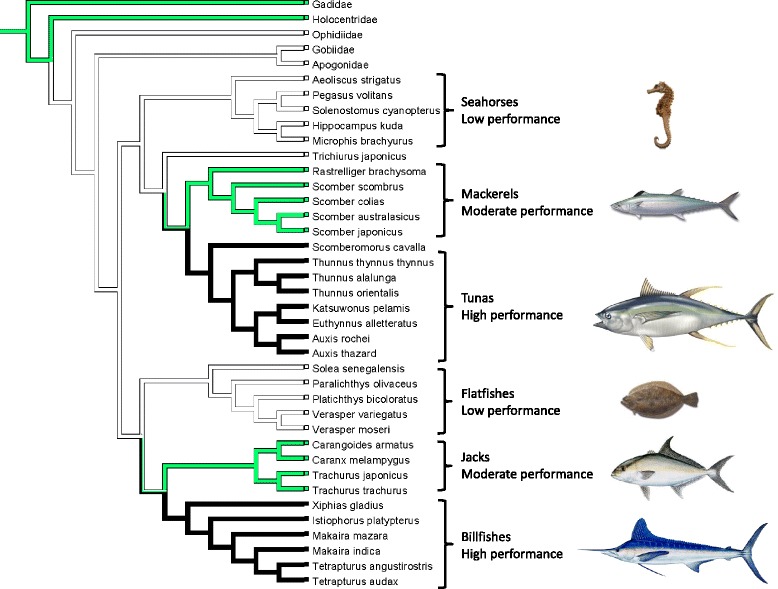
Phylogeny and swimming performance states of fish groups examined in this study. Swimming performance was reconstructed with parsimony (shown), as well as maximum likelihood, which was qualititatively similar, with outgroups from Betancur-R et al. (2013). Branch colors represent performance levels as follows: white, low performance; green, moderate performance; black, high performance

Here, we investigate patterns of adaptation on OXPHOS genes in diverse group of fish taxa with different swimming performance. We hypothesized that positive natural selection would affect OXPHOS efficiency among divergent lineages with long-term differences in ATP demand. Thus molecular adaptation was predicted to be associated with locomotor intensity (speed, duration and frequency). We examined evidence for positive selection on all mitochondrial OXPHOS genes from six fish groups that can be classified into three different swimming performance categories based on general locomotion patterns [22]. Tunas and billfishes represent high performance swimmers, mackerels and jacks represent moderate (or moderate-high) performance swimmers, and flatfishes and seahorses + pipefishes represent low performance swimmers. Pelagic fishes exhibit highly aerobic locomotion with greater endurance than sedentary fishes, and among the pelagic fishes, tunas and billfishes may maintain the highest speeds for the longest duration [22]. Seahorses and pipefishes exhibit much lower frequency, duration, and speed of locomotion, while flatfishes exhibit lower frequency, and sustained speed. These factors as well as their unusual swimming forms and kinematics suggest that much less of their total energy budget is devoted to locomotion, so we categorized them as low-performance swimmers. Recent phylogenetic analyses [43–45] indicate that these taxa are arranged into two monophyletic groups each containing representatives of all three performance classes (Fig. 1). Two previous studies (39, 40) examined positive selection in some high-performance fishes that are included in this study. We extended these studies by including all 13 mitochondrial OXPHOS genes and examined the evidence of positive selection on multiple phylogenetic branches, including six independent fish lineages as well as some ancestral branches. In contrast to previous studies, this approach allowed us to make mitochondrial genome-wide assessments of selection across taxa exhibiting a wide range of locomotor performance. We also inferred functional significance from the position of positively selected amino acid sites in the 3-dimensional structure of specific enzyme complexes. Our results indicate that selection on OXPHOS genes is indeed associated with divergent swimming habits among these fishes. However, selection is heterogeneous over evolutionary time and positive selection is not strictly associated with high performance taxa. Thus we provide new insights on the evolution of swimming diversity in fishes and the adaptation of OXPHOS genes relative to organismal energetic performance on a broad phylogenetic scale.

## Methods

### Phylogeny reconstruction

We acquired sequences of 13 mitochondrial protein genes (all of them encode subunits in OXPHOS) from the representative species of each fish group from National Center for Biotechnology Information (NCBI) GenBank (Additional file 1: Table S1). To complement the mitochondrial genes, we added 7 nuclear genes which are commonly used in fish phylogenies (obtained from NCBI GenBank) or were developed as part of the Fish Tree of Life project [46]. These genes are *rag1* (recombination activating protein 1), *rag2* (recombination activating protein 2), *rhodopsin*, *tmo4c4* (anonymous, see [47]), *zic1* (zinc finger protein 1), *myh6* (myosin, heavy chain six), and *btbd7* (BTB domain containing seven). For species without available sequences, we designed primers (Additional file 1: Table S2) and amplified and sequenced specific genes via standard polymerase chain reaction (PCR). PCR product was sequenced in both forward and reverse directions using ABI BigDye terminator chemistry and an ABI Prism 3130 XL Genetic Analyzer. Sequences have been deposited in GenBank (Additional file 1: Table S3). Particular genes for a few species could not be amplified. In such cases, sequences were obtained from congeners, yielding 19 “chimaeric” individuals. The 20 genes (13 mitochondrial and 7 nuclear) were concatenated for phylogenetic analyses [48].

Maximum likelihood trees (with 1000 bootstrap replicates) were estimated in the program RAxML v.7.0.4 [49]. Bayesian phylogenetic analysis was performed with MrBayes v.3.1 [50, 51]. Swimming performance states were reconstructed from extant taxa with parsimony and likelihood using outgroups from [44] in Mesquite [52].

### Analysis of patterns of natural selection

**Fig. 2:**
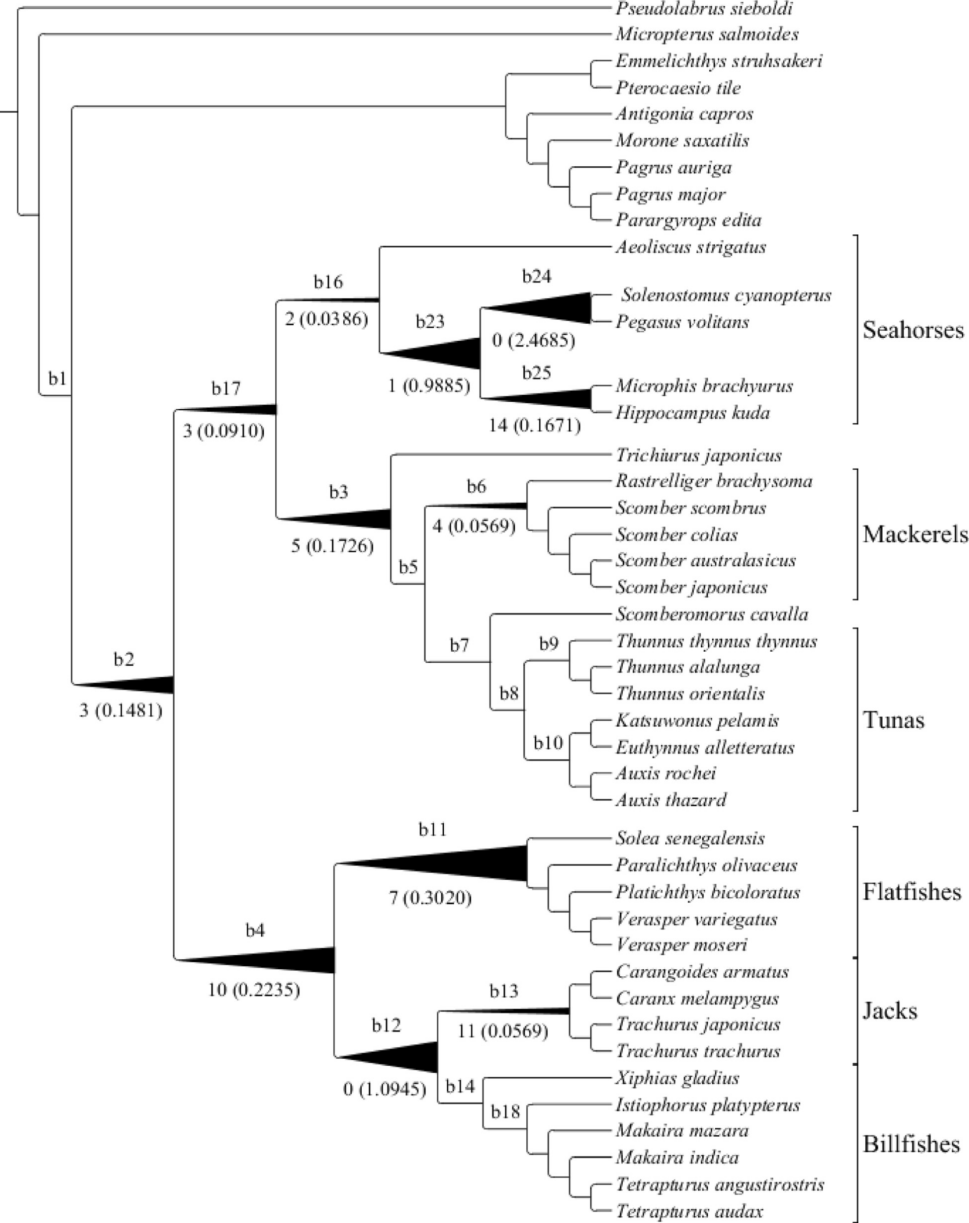
Positive selection analysis on mitochondrial genes on each branch (arbitrary labels, b1-b24, appear above each branch). The number below each branch represents the number of positively selected sites with posterior probability higher than 0.8. The number in parentheses is the product of proportion of sites having *ω* > 1 and *ω*_2_ for that branch, as an indicator of the strength of positive selection. The width of the branches is proportional to the strength of positive selection (because *ω*_2_ for b12 and b24 is extremely high, the width was capped for ease of visualization). Labled branches without values listed have no evidence of positive selection

Sequences of 13 mitochondrial OXPHOS genes were used to examine patterns of natural selection. We first ran a series of random sites models (M0, M1a, M2a, M3, M7, M8a and M8) implemented in Codeml of PAML v.4.7 [53]. Likelihood ratio tests (LRTs) were conducted on the likelihood values produced by specific pairs of models: M3 vs. M0, M2a vs. M1a, M8 vs. M7, and M8 vs. M8a. Next we ran branch-site models on designated branches with three different starting *ω* values (0.1, 1.0, and 4.3). We had no prior knowledge of which branch(es) could have experienced positive natural selection (except for branches leading to tunas and billfishes); therefore, we designated one branch (from b1-25 in Fig. 2) as the foreground branch in each test. LRTs were performed to determine if the more complex model A is significantly better than the null model. In the branches that showed evidence of positive selection, we used Bayes empirical Bayes (BEB) to calculate the probability of amino acid sites are under positive selection.

The 13 mitochondrial gene sequences were also analyzed using TreeSAAP [54], which measures the selective influences on 31 structural and biochemical amino acid properties, and performs goodness-of-fit and categorical statistical tests. We used a sliding window size of 20 amino acids (Additional file 1: Figure S2). Because of the large number of sites under positive selection, we only show sites with significance when its *p* <0.001 for clarity. Then we used a sliding window size of 1 and one amino acid site is defined as being positively selected as long as one of the 31 properties showed significance.

### Mapping positively selected amino acid sites onto 3-dimensional (3D) crystal protein structure

The *MT-ND* genes that encode subunits in OXPHOS complex I are considered highly conserved between prokaryotes and vertebrates [55]. We inferred the potential function of positively selected amino acid sites belonging to *MT-ND* subunits by mapping them onto the 3D crystal structure of *Thermus thermophiles* (PDB ID: 4HEA), a Gram negative eubacterium [56]. We mapped the positively selected sites of *MT-CYB* onto chicken bc_1_ complex C chain (PDB ID: 1BCC) crystal structure because a study suggests the structure of the catfish *MT-CYB* protein resembles that of chickens [57]. Similarly, we mapped the positively selected sites onto bovine *MT-CO1-3* subunits 3D structure (PDB ID: 1OCC) because of the highly conserved structure from bacteria to bovine [58, 59]. All the mapping was conducted using Geneious pro (v.7.0).

## Results

### Phylogeny and ancestral state reconstruction

Both maximum likelihood and Bayesian analyses generated the same topology (Fig. 2) regardless of partition scheme with relatively high bootstrap support and posterior probabilities (see Additional file 1: Figure S1). The recent comprehensive phylogenetic analysis of [44] resolved the phylogeny of bony fishes to the level of taxonomic family, while a study by [45] focused on relationships of several pelagic fish groups. Our results are consistent with both of these studies for the taxa in common to each. Specifically, we recovered two monophyletic groups, each containing taxa with low, moderate, and high swimming performance. In one group, the tunas and mackerels (Scombriformes) are sister to the seahorses and pipefishes (Syngnathiformes), while the series Carangaria contains the billfishes (Istiophoriformes), jacks (Carangiformes), and flatfishes (Pleuronectiformes) [44] (see also Phylogenetic Classification of Bony Fishes--Version 3: www.deepfin.org).

Swimming performance states were reconstructed from extant taxa with parsimony and likelihood using outgroups from [44]. These results of reconstructed swimming performance suggest that the ancestors of each major group were low performance swimmers (Fig. 1), although incomplete taxon sampling of related groups could mislead the reconstructions. Even if the ancestors were moderate performance swimmers, it is clear that high performance swimming evolved independently in the tunas and billfishes.

### Analysis of positive selection

Positive selection under site models cannot identify particular branches where positive selection has occurred, but they can detect positive selection among sites where it occurs as long-term trends or among multiple branches separated on the tree. A significant difference was detected between model M0 and M3, which suggests *ω* is variable among sites (Table 1). We used three model pairs to test for positive selection: M1a vs. M2a, M7 vs. M8, and M8a vs. M8. Neither M2a-M1a nor M8a-M8 showed significant difference. However, M8-M7 showed a significant difference, suggesting some positive selection signal on certain sites somewhere on the tree.

**Table 1:** Results of PAML Random Sites models

Model	lnL	Parameters^a^			Null	LRTs	*p*
		*ω*_*0*_/*p*	*ω*_*1*_/*q*	*ω*_*2*_/ *ω*_*p*_			
M0	−191327.6225	0.03421					
M1a	−189631.0183	0.02838 (94.9 %)	1 (5.1 %)				
M2a	−189631.0190	0.02838 (94.9 %)	1 (5.1 %)		M1a	0	
M3	−184769.7796	0.00609 (72.6 %)	0.11443 (27.4 %)	79.11600 (0)	M0	13115.6857	**
M7	−184829.5771	0.22705	4.62358				
M8a	−184813.0237	0.23248	5.02542	1.00000			
M8	−184813.0237	0.23248	5.02544	1.00000	M7	33.1068	**
					M8a	0	

*lnL*; log likelihood;

LRTs represents the likelihood ratio tests; 2 * (lnL(Model) – lnL(Null)).** represents *p* < 0.01

^a^ *ω* values of each site class are shown for models M0-M3 (*ω*_*0*_ – *ω*_*2*_) with the proportion of each site class in parentheses. For M7-M8, the shape parameters, *p* and *q*, which describe the beta distribution are listed

Table 2 lists the resulting likelihood values, likelihood ratio tests (LRTs), and estimated model parameters for each branch examined under branch-site models. We performed a false discovery rate analysis [60] on these results where we performed 20 tests and recovered 12 positive results. All 12 positive results had a q value of 0.03 or less, and the probability of a false positive among them is 0.36. Because this is less than 1.0, no false positives are expected. The number of amino acid sites on each branch inferred to be under positive selection via Bayes Empirical Bayes is provided Additional file 1: Table S4 (the identity of these sites and more details are provided in Additional file 1: Table S5). These results showed substantial differences in natural selection on lineages leading to taxa with different swimming performance. We found significant evidence of positive selection on the ancestral lineages of all three performance categories (branches b2, b17, b3, and b4 in Fig. 2), lineages leading to moderate-performance swimmers (b6 and b13), and low-performance swimmers (b16, b23, b24, b25, and b11). Conversely, strong purifying (negative) selection was identified on lineages of high-performance swimmers (b7, b8, b9, b10, b14, and b18). Fourteen sites that appeared to be positively selected occur on multiple branches; however, they are not associated with branches leading to a particular performance group (see details in Additional file 1: Table S5).

**Table 2:** Results of PAML branch-site model analysis

Branch	Model A	Null model	LRTs	Site class	0	1	2a	2b
b1	−189631.0183	−189631.0183	0	Proportion	0.94985	0.05015	0.00000	0.00000
				Foreground *ω*	0.02838	1.00000	1.00000	1.00000
b2	−189619.8114	−189623.914	8.2052^**^	Proportion	0.94260	0.04932	0.00769	0.00040
				Foreground *ω*	0.02825	1.00000	18.30494	18.30494
b3	−189710.2567	−189714.0692	7.6250^**^	Proportion	0.92062	0.04856	0.02927	0.00154
				Foreground *ω*	0.02811	1.00000	5.60205	5.60205
b4	−189591.2640	−189598.5732	14.6183^**^	Proportion	0.92700	0.04823	0.02355	0.00123
				Foreground *ω*	0.02799	1.00000	9.01795	9.01795
b5	−189728.0764	−189729.5338	2.9148	Proportion	0.91976	0.04869	0.02997	0.00159
				Foreground *ω*	0.02824	1.00000	292.74200	292.74200
b6	−189719.7435	−189722.2222	4.9573^*^	Proportion	0.93956	0.04997	0.00994	0.00053
				Foreground *ω*	0.02824	1.00000	5.43888	5.43888
b7	−189738.4458	−189738.4458	0	Proportion	0.94477	0.05030	0.00468	0.00025
				Foreground *ω*	0.02845	1.00000	1.00000	1.00000
b8	−189738.5873	−189738.5873	0	Proportion	0.94946	0.05054	0.00000	0.00000
				Foreground *ω*	0.02848	1.00000	1.00000	1.00000
b9	−189738.5873	−189738.5873	0	Proportion	0.94946	0.05054	0.00000	0.00000
				Foreground *ω*	0.02848	1.00000	1.00000	1.00000
b10	−189738.5873	−189738.5873	0	Proportion	0.94946	0.05054	0.00000	0.00000
				Foreground *ω*	0.02848	1.00000	1.00000	1.00000
b11	−189613.4925	−189622.0967	17.2084^**^	Proportion	0.94688	0.04932	0.00362	0.00019
				Foreground *ω*	0.02831	1.00000	79.27458	79.27458
b12	−189615.9415	−189619.0732	6.7338^**^	Proportion	0.92922	0.04889	0.02080	0.00109
				Foreground *ω*	0.02820	1.00000	999.00000	999.00000
b13	−189610.5139	−189616.7321	12.4364^**^	Proportion	0.94411	0.04933	0.00624	0.00033
				Foreground *ω*	0.02821	1.00000	8.65776	8.65776
b14	−189734.9950	−189736.0673	2.1446	Proportion	0.94724	0.05043	0.00221	0.00012
				Foreground *ω*	0.02842	1.00000	4.83612	4.83612
b16	−189624.9748	−189628.011	6.0725^**^	Proportion	0.94529	0.04967	0.00479	0.00025
				Foreground *ω*	0.02827	1.00000	7.66238	7.66238
b17	−189621.7633	−189627.8443	12.1622^**^	Proportion	0.94863	0.04955	0.00173	0.00009
				Foreground *ω*	0.02835	1.00000	336.12589	336.12589
b18	−189738.5873	−189738.5873	0	Proportion	0.94945	0.05055	0.00000	0.00000
				Foreground *ω*	0.02848	1.00000	1.00000	1.00000
b23	−189618.7575	−189625.3146	13.1142^*^	Proportion	0.93161	0.04861	0.01879	0.00098
				Foreground *ω*	0.02817	1.00000	999.00000	999.00000
b24	−189578.5777	−189597.2838	37.4121^**^	Proportion	0.90294	0.04769	0.04689	0.00248
				Foreground *ω*	0.02828	1.00000	999.00000	999.00000
b25	−189591.1234	−189603.6533	25.0598^**^	Proportion	0.93264	0.04910	0.01735	0.00091
				Foreground *ω*	0.02815	1.00000	9.15193	9.15193

Estimated likelihood values under model A (allowing positive selection) and the null model (no positive selection), likelihood ratio tests (LRTs), and estimated parameters of model A. LRT critical values 3.84 at *p* = 0.05 (*) and 5.99 at *p* = 0.01 (**). Branches are as identified in Fig. 2

The above tests assess the strength of the evidence for positive selection rather than the strength of selection itself. An indicator of the strength of positive selection on a branch can be obtained from the product of the proportion of sites having *ω* > 1 and the estimated *ω* value for those sites. In Fig. 2, the branch width is shown proportional to the strength of positive selection based on this measure (except for b12, b23 and b24 which had extremely high *ω* and a cap of *ω* = 50 is used). Among those branches harboring sites under positive selection, branches ancestral to more than one swimming category (b2, b3, b4, b12, b17) and branches leading to low-performance fishes (b11 and b23) exhibited greater selection than branches leading to moderate-performance fishes (b6 and b13), and substantially more than any branches associated with high-performance fishes. This is counter to the notion that high performance swimmers will have been most strongly influenced by positive selection for enhanced OXPHOS performance assuming enhanced OXPHOS efficiency is the major contributor to high performance. However, there are alternative contributors to high performance such as increased mitochondrial density per tissue mass, more closely packed inner mitochondrial membrane cristae, increased metabolic enzyme activity, increased expression of genes involved in a number of biological pathways such as glycolysis, protein biosynthesis, and cytoskeletal structure. Under any of the later scenarios, an increase in OXPHOS efficiency might not be necessary for high performance.

The physico-chemical properties analysis as implemented by TreeSAAP [54] does not provide inferences about particular branches on the phylogeny, but does provide information about changes of particular amino acid sites across the whole tree. A large number of amino acid sites were identified as having substitutions with significant physico-chemical differences; presented in Additional file 1: Figure S2 and Table S4. A mitochondrial genome-wide (only the protein coding genes) sliding window analysis (window size = 20 amino acids) revealed that the vast majority of windows contained substitutions that exhibited between 0 and 15 properties with significant differences (Additional file 1: Figure S2). Windows with substitutions exhibiting between 15 and 30 significant properties occurred at much lower frequency. Although windows with the greatest number of significant property differences were observed in the genes for *MT-ND1*, *MT-CO1*, *MT-ND4* and *MT-CYB*, there were no other apparent patterns of variation in the distribution of such sites across the genome (Additional file 1: Figure S2). Positively-selected sites identified by both methods are summarized in Additional file 1: Table S4, S5.

The two lineages, seahorses and flatfishes, on which positive selection was most pronounced, have also experienced extraordinary morphological evolution [61, 62]. Thus, it is possible that the positive selection signal detected on mitochondrial OXPHOS genes in these lineages is unrelated to energy demands, but is simply a consequence of rapid genomic evolution in these groups. The probability of nucleotide change is indeed higher in these two lineages than for the other major groups as indicated by five nuclear genes that are not directly involved in OXPHOS system (*rag1*, *rhodopsin*, *tmo4c4*, *mhy6*, and *zic1*). However, a positive selection signal was found for only two genes, *rag1* and *rhodopsin*, on one branch leading to flatfishes (LRTs = 72.53, *ω* = 50.88, three sites 199, 425, and 605 under selection with bayes empirical bayes (BEB) probability of 0.684, 0.501, and 0.958, respectively). Because there was no consistent pattern of selection on these non-OXPHOS genes, it is suggested that the positive selection detected on mitochondrial genes does not reflect a genome-wide pattern of divergence but may be related to adaptation of OXPHOS efficiency.

### Structural position of positively selected sites

The position of particular amino acids in the tertiary and quaternary structure of a protein may allow inferences about the function of individual residues. In particular, those sites near the catalytic core or other functionally important regions, or those in physical proximity (likely to interact with) other amino acids, would seem most likely to influence protein function.

**Fig. 3:**
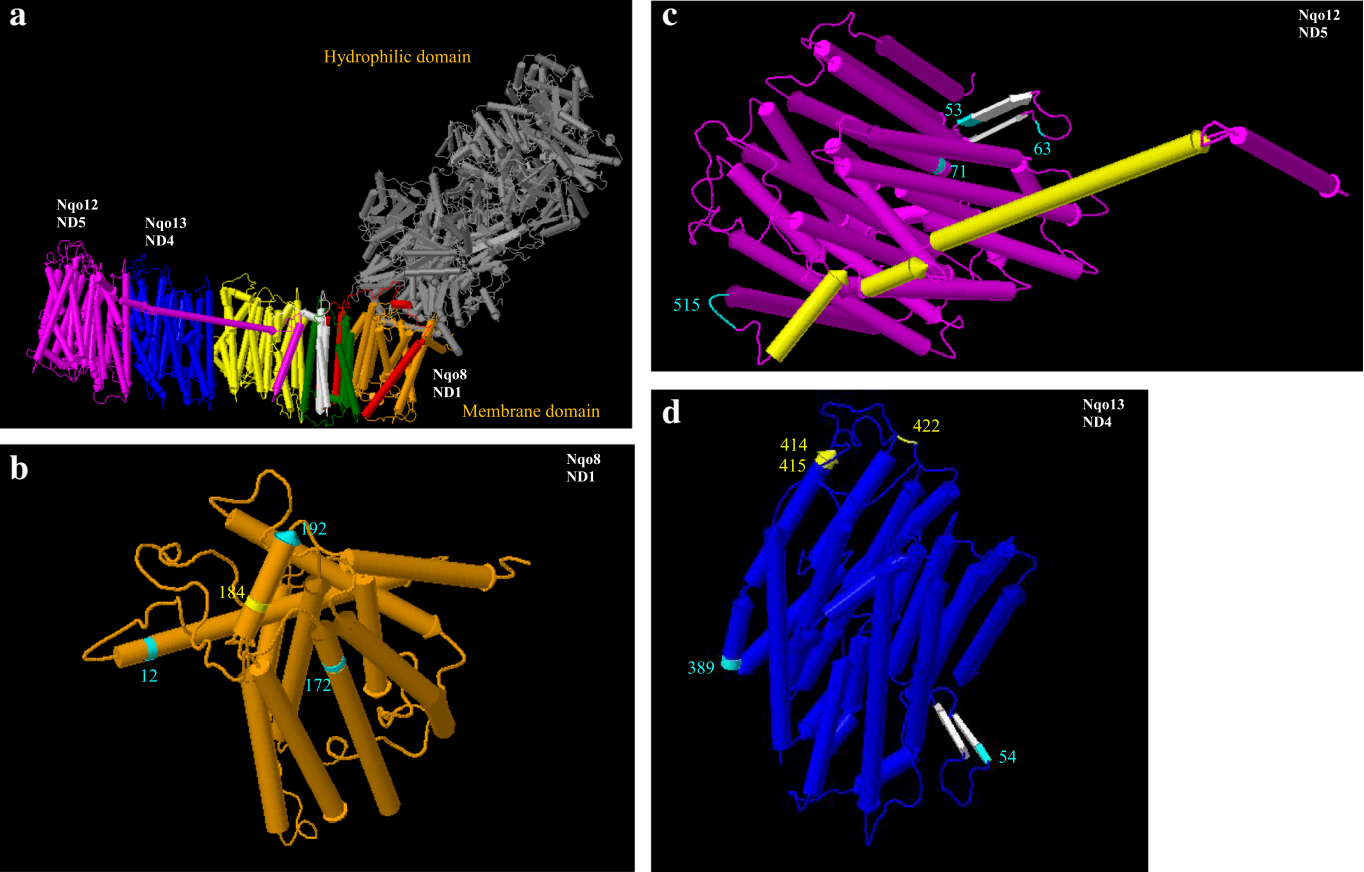
Crystal structure of the entire respiratory complex I at 3.3 Å (PDB: 4HEA) from *Thermus thermophiles* front view (**a**). Mitochondrial gene encoded subunits include Nqo8 (*MT-ND1*) (orange) (**b**), Nqo14 (*MT-ND2*) (yellow), Nqo7 (*MT-ND3*) (red), Nqo13 (*MT-ND4*) (blue) (**d**), Nqo11 (*MT-ND4L*) (white), Nqo12 (*MT-ND5*) (magenta) (**c**), and Nqo10 (*MT-ND6*) (green) subunits. Amino acid sites in cyan are those with evidence of positive selection in this study. Structure in gray indicates the nuclear-gene encoded hydrophilic domain

Complex I performs the first step and is the largest and most complicated enzyme complex in the OXPHOS pathway. It catalyzes the transfer of two electrons from NADH to ubiquinone (Q), coupled to the translocation of four protons across the inner mitochondrial membrane. It is also a major source of reactive oxygen species in mitochondria. Complex I exhibits an L-shaped architecture with a membrane arm and a hydrophilic peripheral arm that protrudes into the mitochondrial matrix (Fig. 3a). The membrane arm consists of 7 core mitochondrial NADH dehydrogenase (*MT-ND*) gene encoded subunits.

Site 12, one of the sites identified in subunit ND1 as being positively selected (Fig. 3b), is included in the region associated with MELAS (mitochondrial encephalomyopathy, lactic acidosis, and stroke-like episodes)/DEAF enhancer/hypertension [63] and sudden infant death [64]. The other positively selected sites, 172 and 192, are in the alpha helix close to a critical site, 184, that is associated with adult onset dystonia [65] (in yellow in Fig. 3b).

Subunit ND5 contains an unusual structural element, the helix HL (see enlarged Fig. 3c, indicated in yellow), that extends nearly the entire length of the membrane domain and coordinates conformational changes. On the opposite side of the membrane domain, a series of β-hairpins (βH element) (indicated in white in Fig. 3c, d) from neighboring subunits contribute to conformational changes and stability of the complex. Positively-selected sites include 515 [ND5], which is adjacent to helix HL; 53 [ND5] and 54 [ND4] are in βH elements; 63 [ND5] is in the seven-residue loop connecting two βH elements; 86 [ND2] (not shown here) and 71 [ND5] are in regions (including residues 83 [ND2] and 88 [ND5]) demonstrated to have significant negative effects on function [66].

In addition to the sites listed above, there are several sites that appear to be under positive selection, yet their location in the molecular structure does not provide any clear suggestion of functional significance. These residues could be involved in stabilizing the tertiary or quaternary structure of the various multi-subunit complexes or facilitate their assembly. These include residues 199 [ND5], 269 [ND5], 425 [ND5], 515 [ND5], 600 [ND5], 20 [ND4], 86 [ND2], 168 [ND2], 359 [ND2], 360 [ND2], 408 [ND2], 23 [ND4L], 4 [ND6], 8 [ND6], 11 [ND6], 90 [ND6], 138 [ND6], 94 [ND3] and 98 [ND3] (not shown in figures for clarity).

**Fig. 4:**
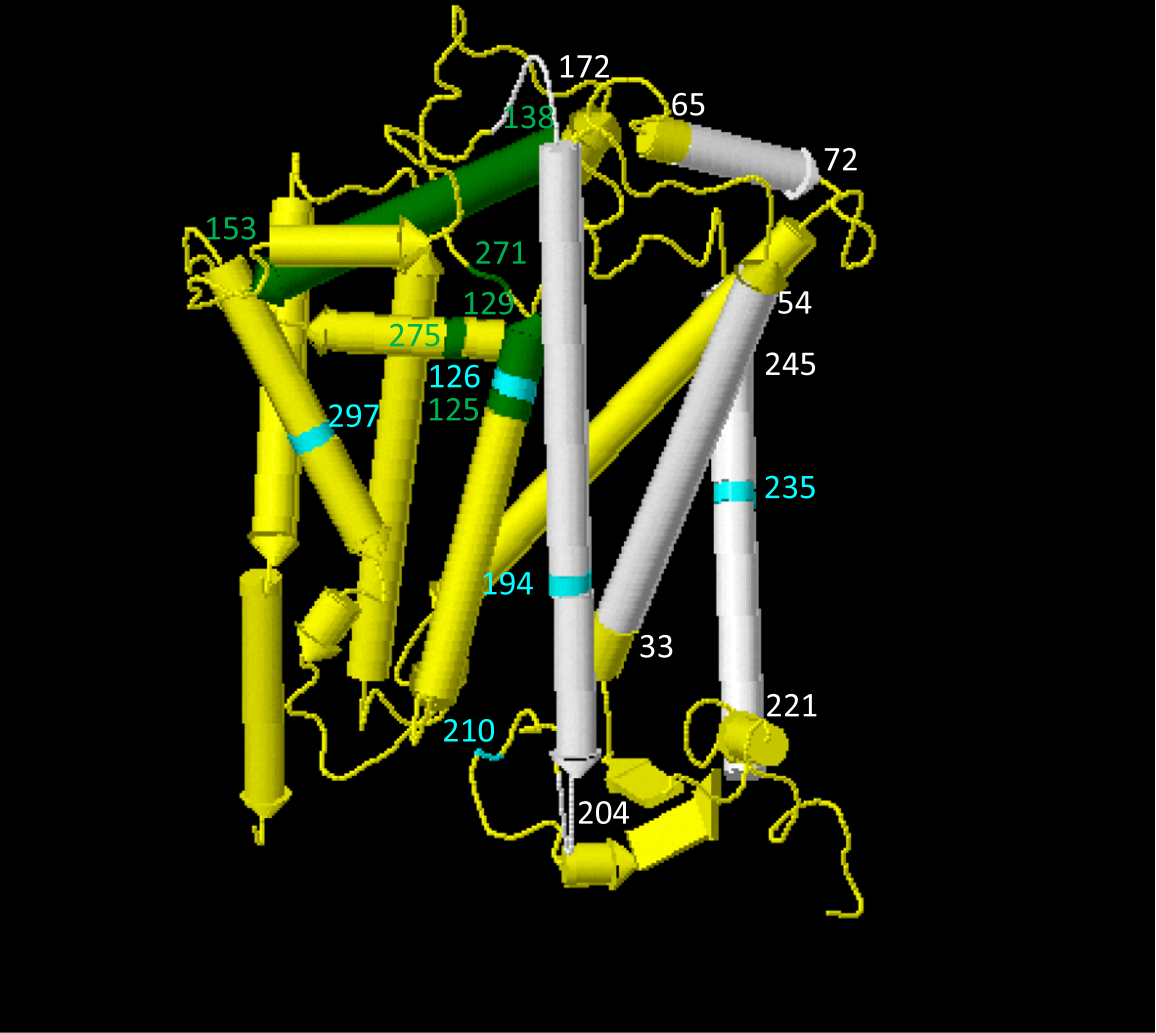
Crystal structure of the entire respiratory complex III cytochrome b at 3.16 Å (PDB: 1BCC) from chicken (*Gallus gallus*). White indicates antimycin-binding cavity formed by helices A (residues 33–54), D (172–204), E (221–245), and the amphipathic surface of helix a (65–72). Green indicates stigmatellin- and myxothiazol-binding pocket formed by residues 271, 275, 125–129, and 138–153. Cyan residues are those identified as positively selected amino acid sites in this study

Complex III is an intermediate component of the respiratory chain, which transfers electrons from reduced ubiquinone to cytochrome c, coupled to proton translocation across the mitochondrial membrane [67]. The *MT-CYB* gene encoded cytochrome b forms the active redox center: a cavity surrounded by the transmembrane helices A (residues 33–54), D (172–204), and E (221–245), and the amphipathic surface helix a (65–72) (indicated in white in Fig. 4). Residues 221 and 194 are also close enough to contact the active site inhibitor [68]. The positively selected sites 194 and 235 (indicated in cyan in Fig. 4) are within these regions, suggesting important functional effects.

The pocket bound by stigmatellin, one OXPHOS inhibitor, is formed by the end of helix C, the helix cd1, the helix ef linker, and the end of helix F. Specific residues of known importance include 271, 275, 125–129, 138–153 (indicated in green in Fig. 4). The positively selected sites 126 and 297 are close to this area.

**Fig. 5:**
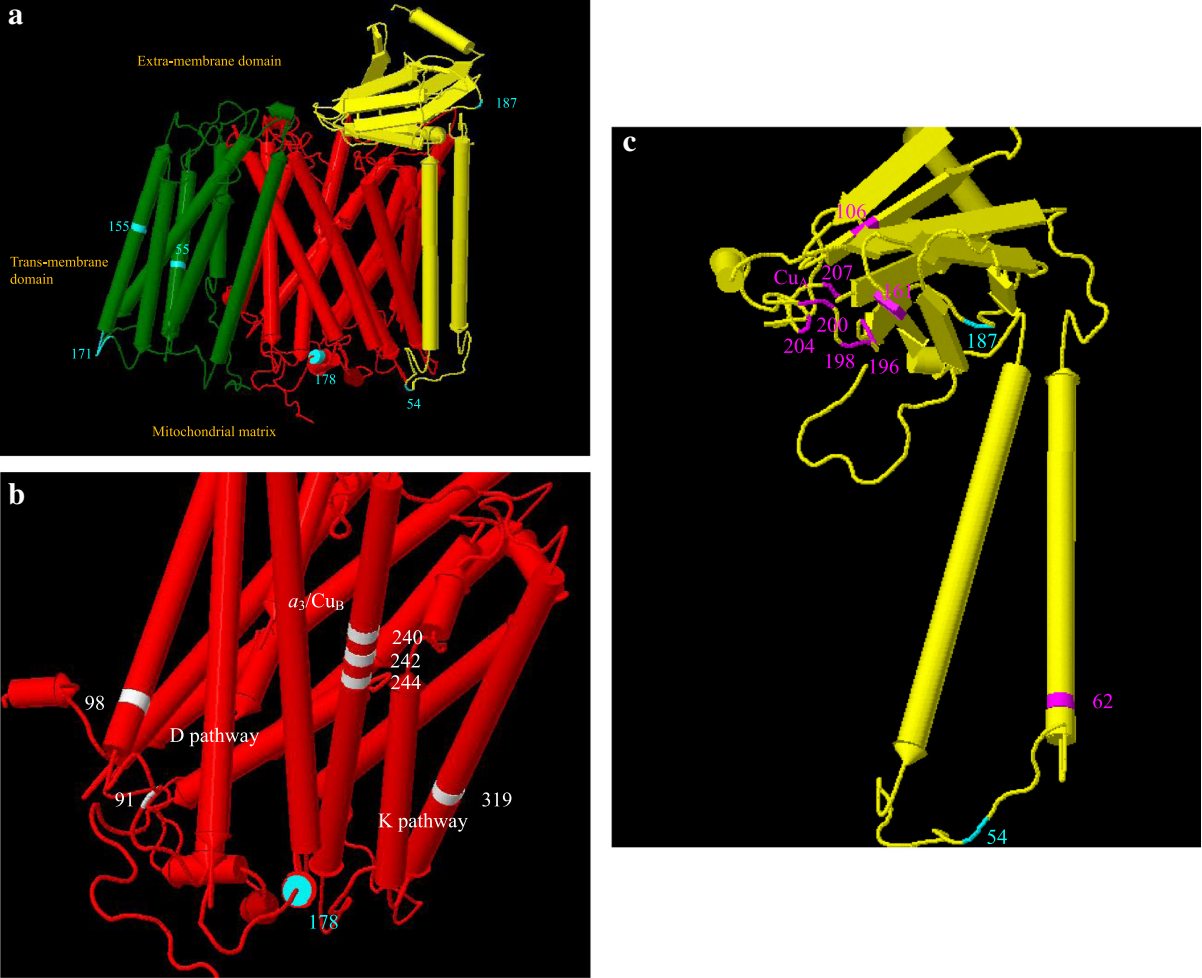
Structure of mitochondrial DNA encoded *MT-CO1* (*red*) (also see in 5**b**), *MT-CO2* (*yellow*) (also see in 5**c**), and *MT-CO3* (*green*) subunits from bovine heart cytochrome c oxidase at 2.8 Å (PDB: 1OCC) (5**a**). Amino acid sites in cyan are positively selected sites detected in this study (5**a**). White indicates key residues in proton translocation pathways: D-pathway and K-pathway in subunit I. Magenta indicates key residue for electron to enter and CuA liganded residues in subunit II

Complex IV is the terminal component of the respiratory chain, in which electrons received from cytochrome c reduce molecular oxygen to water and protons are pumped into the intermembrane space. Six positively selected sites were identified in complex IV: 178 in subunit COX1, 54 and 187 in subunit COX2, and 55, 155, and 171 in subunit COX3 (Fig. 5a).

Subunit I (encoded by *MT-CO1* gene) is largely embedded in the membrane with three redox centers: heme a, heme a_3_, and Cu_B_. One of the Cu_B_ liganded residues is 240. It has been suggested that residue 244 and 240 are close enough for binding interaction, thereby forming an unusual cross-link structure [69]. One positively selected site 178 is very close to 244 and 240 (Fig. 5b). Two proton translocation pathways, D-pathway and K-pathway, are identified in subunit I. Three key residues of D-pathway 91, 98, and 242, and one key residue of K-pathway 319 are well defined from mutagenesis studies [69] (key residues are indicated in white in Fig. 5b). The positively selected site 178 (indicated in cyan in Fig. 5b) is close to (within 30 Å) the four key residues and could potentially affect proton translocation.

Subunit II (encoded by *MT-CO2* gene) has two trans-membrane helices and a hydrophilic beta strand extending to the extra-membrane domain, housing the Cu_A_ center. The first residue crucial for electron to enter the oxidase complex cytochrome c is 106 (indicated in magenta in Fig. 5c). The other Cu_A_ liganded residues identified through mutagenesis include 196, 200, 198, 161, 204, and 207 (indicated in magenta in Fig. 5c). One positively selected site 187 is very close to the Cu_A_ center and the other positively selected site 54 is in a linking strand between two helices of the entry site which includes critical residue 62 (indicated in cyan in Fig. 5c).

Subunit III (encoded by *MT-CO3* gene) is fully embedded in the membrane domain. No key residue of this subunit has been identified through mutagenesis. However, it has been shown that this subunit stabilizes the integrity of the binuclear center in subunit I; for example, when this gene is deleted, only a partially assembled complex results [69].

The sites described above as having potential functional significance were more or less uniformly distributed among branches of the tree and showed no association with particular swimming performance groups. Many sites exhibited evidence of being under positive selection yet did not appear in structural locations that would suggest special functional importance. However, the interaction between amino acids among different proteins or among sites in the same protein is known to have significant functional effects (e.g., [70, 71]). Such interactions could affect protein assembly, stability or specific function. We used 4 Å as the nominal upper limit for weak interactions between amino acid sites as described in [72]. None of the sites identified here as under positive selection were found to be within 4 Å of any other amino acid sites.

## Discussion

We examined evidence for adaptation of mitochondrial OXPHOS genes in fishes with different swimming performance. Selection was investigated for specific amino acids sites across the whole phylogenetic tree for these species, as well as for amino acid sites on individual branches of the tree. The results show a strong signal of positive selection on branches in the ancestral parts of the tree, and branches leading to low- and moderate-performance swimmers. However, no evidence of positive selection was observed within clades of the high-performance tunas or billfishes. We did not observe a disproportionate effect of selection on any particular gene as all genes exhibited some positively selected sites but this varied across branches of the tree. We also show that many of the sites identified as being under positive selection occur in structural regions where they may have effects on OXPHOS function. Positively selected sites in other regions may also have functional significance, but their potential effects are less clear.

Our results show the strongest signal of positive selection on branches leading to the lowest performance fishes, while purifying selection was identified on the branches of high performance fishes. This result is in strong contrast to previous studies that have exclusively examined the hypothesized association of positive selection on OXPHOS with increased energetic demands required by higher organismal performance (reviewed in [16]). Our results suggest that a fairly efficient OXPHOS system had evolved under positive selection in the ancestors of the two major groups. Selective modification of OXPHOS on branches leading directly to tunas and billfishes may have further facilitated the evolution of high performance swimming. But once very high performance swimming had evolved in these lineages, purifying selection appears to have predominated on the OXPHOS system as it existed at that time. Conversely, substantial modification due to positive selection occurred in the lower performance lineages of flatfishes and the seahorse + pipefish group. The moderate-high performance jacks and mackerels exhibited moderate-high conservation, with limited positive selection. Thus, the strength of the positive selection signal is inversely proportional to swimming performance within both of these taxonomic groups.

A common expectation is that positive selection should lead to enhanced organismal performance. Moreover, it might be expected that positive selection will lead to increased functional efficiency of OXPHOS in response to the increased ATP demands associated with enhanced performance. In the absence of information on the exact functional significance of individual substitutions, the effects of positive selection to increase or decrease OXPHOS efficiency in these taxa remains unknown. However, our results do not match simple expectations. Positive selection appears to be heterogeneous, fluctuating over phylogenetic time scales, with no simple relationship between the strength of selection and organismal performance. However, one clear pattern is that there is strong functional constraint (negative selection) in these high performance systems and directional selection on these lower performance groups.

Our results appear to contrast with previous studies where positive selection or evolutionary rate variation was found on lineages leading to organisms with high ATP demands. For example, *MT-CO1* [73] and *MT-CO2* [74] exhibited accelerated d_*N*_ in the lineage leading to hominids that appears to be associated with increased brain size. Grossman et al. [75] found accelerated rate variation for *MT-CO4*, a nuclear gene in OXPHOS complex IV in catarrhine ancestors of hominids in the period between 18 and 40 Mya and then decelerated in the descendant hominid lineages. On the lineage leading to bats, the only mammals capable of powered flight, eight OXPHOS genes were found to have undergone positive selection [18]. Foote et al. [76] found two positively selected amino acid sites, which could influence overall metabolic performance, in the mitochondrial genes of killer whales (*Orcinus orca*). In addition, the *MT-ND2* and *MT-ND5* genes of highly migratory Pacific salmon exhibit evidence of positive selection [41].

Our results are consistent with previous studies of the high-performance taxa examined here. Dalziel et al. [39] examined one mitochondrial OXPHOS gene (*MT-CO2*) on several branches among high-performance fishes, including billfishes and tunas. They found *ω* was not increased in lineages leading to the tunas but was significantly increased in the lineage preceding the billfish (including several amino acid sites). However, the phylogeny used in [39] does not include the flatfish or seahorse clades, which are now recognized as close relatives of billfishes and tunas, respectively. Little et al. [40] examined three mitochondrial OXPHOS genes (*MT-CO1, 2, and 3*) on the single lineage leading to billfishes and found positive selection along that lineage when flatfishes were excluded but no selection was detected when flatfishes were included. The latter result is consistent with our findings and as suggested by Little et al. [40] emphasizes the importance of dense phylogenetic sampling for the analysis of positive selection. We extended these two studies by examining 10 additional mitochondrial OXPHOS genes in the high-performance taxa, and also by explicitly investigating selection on related low performance taxa.

With respect to negative selection on high performance fishes, we speculate that once a reasonably efficient OXPHOS system evolved it may have become difficult to change in the high performance groups. The high performance system might be expected to have a much lower tolerance for non-synonymous substitutions as most (even slight) changes would be likely to have negative functional effects. Conversely, lower performance swimmers may have a much broader tolerance for non-synonymous substitutions because the OXPHOS system is under much lower performance demands. We envision the high performance swimmers as occupying a local optimum on a fitness landscape [77], but their OXPHOS system is so fine-tuned that substitutions that would allow them to cross fitness valleys and reach higher peaks could be strongly deleterious in the short term. On the other hand, lower performance fishes might readily cross such valleys without significant fitness costs because OXPHOS efficiency will be less critical and they may then climb other fitness peaks due to positive selection.

It is also possible that organismal fitness in taxa with low energy demands may be increased by a modified OXPHOS regulatory system or even a reduction in OXPHOS efficiency. In such cases, we would expect to see evidence of positive selection on OXPHOS genes in these taxa. Because there may be trade-offs between OXPHOS rate or efficiency and deleterious effects, reducing OXPHOS efficiency may be adaptive in systems where ATP demand is chronically low. For example, maintenance of a strong chemiosmotic gradient in organisms with low ATP demand may cause increased production of reactive oxygen species (ROS) [38] leading to oxidative stress [78]. Therefore reduction of the H^+^/2e ratio (increased slippage) due to altered protein structures could be adaptive in low performance species. OXPHOS regulation is highly complex and involves mechanisms independent of structural OXPHOS proteins. Elevated expression of OXPHOS genes and others involved in aerobic respiration could clearly increase aerobic capacity in the absence of selection on specific OXPHOS variants. However, it is clear that positive selection acts on OXPHOS proteins and the effects of positive selection are frequently associated with the evolution of differences in ATP demand.

There are other issues that could affect the efficiency of ATP generation of OXPHOS in high-performance tunas and billfishes. Tunas and billfishes exhibit high metabolic rates to generate ATP to support their high swimming performance (thus high OXPHOS efficiency). However, they are among the few endothermic species of fishes [43, 79, 80] that generate great amount of heat with high metabolic rates (thus reducing ATP generation). Thus, depending on the extent to which body heat is derived from OXPHOS uncoupling, there might be trade-offs between ATP generation and heat generation in these endothermic high-performance fishes. In addition, it has been shown that the heat through mitochondrial proton leak differs among organisms and even differs in cells and tissues in the same organism [38]. It would be helpful to have empirical data about the P/O ratio (how many ATP molecules made from ADP for each oxygen atom consumed), the H^+^/O ratio (how many protons do mitochondria pump from matrix into inter-membrane space for each oxygen atom consumed), and the H^+^/ATP ratio (how many protons flow back to the matrix for each ADP molecule phosphorylated to ATP) in these fish species.

High mitochondrial respiration capacity can be achieved through means other than modification of OXPHOS efficiency via genetic variation, such as increased mitochondrial density per tissue mass, more closely packed inner mitochondrial membrane cristae, increased metabolic enzyme activity, increased expression of genes involved in a number of different but related biological pathways such as glycolysis, the Krebs cycle, and fatty acid metabolism. In addition, swimming performance, as in our qualitatively defined groups, may have arisen due to many different morphological and physiological characteristics beyond OXPHOS function. We do not assert that all variation in swimming performance is due to OXPHOS variation, only that some unknown fraction of the performance variation is positively associated with natural selection. A number of recent studies have presented evidence for direct effects of OXPHOS variation on organismal fitness, many with an identified mechanistic basis [81–86]. While these studies employ tractable model organisms such as *Drosophila*, they provide strong evidence that OXPHOS variation affects organismal performance and fitness in the wild.

In conclusion, we found that patterns of natural selection on mitochondrial OXPHOS genes are complex among fishes with different swimming performance. The type and direction of selection are heterogeneous through evolutionary time and vary in ways that would not be readily predicted based solely on organismal performance. The most striking result was extensive positive selection on low-performance swimmers. Although examination of the most recent lineages indicates that positive selection is inversely proportional to organismal performance, the broader pattern indicates a complex interplay between organismal adaptations, ATP demand and OXPHOS function through evolutionary time.

### Availability of supporting data

The data sets supporting the results of this article are included in its additional files.

## Additional files

Additional file 1: Table S1. Species list with mitochondrial genome accession number from NCBI. **Table S2.** PCR primers used for nuclear markers used in this study. **Table S3.** GenBank accession number of nuclear gene (rag1, rag2, tmo4c4, zic1, myh6, and btbd7) sequences of studied species. **Figure S1.** Phylogeny of six fish groups based on Bayesian and maximum likelihood methods. **Figure S2.** Summary of selection on all mitochondrial genes. **Table S4.** Number of amino acid sites showing positive selection signals in each mitochondrial gene on relevant branches. **Table S5.** Positively selected amino acid sites for each gene on various branches. (DOCX 351 kb)

## Abbreviations

MT-CO1: Mitochondrially encoded cytochrome c oxidase I
MT-CO2: Mitochondrially encoded cytochrome c oxidase II
MT-CO3: Mitochondrially encoded cytochrome c oxidase III
MT-ND1: Mitochondrially encoded NADH dehydrogenase 1
MT-ND2: Mitochondrially encoded NADH dehydrogenase 2
MT-ND3: Mitochondrially encoded NADH dehydrogenase 3
MT-ND4: Mitochondrially encoded NADH dehydrogenase 4
MT-ND4L: Mitochondrially encoded NADH dehydrogenase 4 L
MT-ND5: Mitochondrially encoded NADH dehydrogenase 5
MT-ND6: Mitochondrially encoded NADH dehydrogenase 6
MT-ND: Mitochondrial I: NADH dehydrogenase (ubiquinone) subunits
MT-CYB: Mitochondrially encoded cytochrome b
MT-ATP6: Mitochondrially encoded ATP synthase 6
MT-ATP8: Mitochondrially encoded ATP synthase 8
