# Supplementary material for: Heterogeneous natural selection on oxidative phosphorylation genes among fishes with extreme high and low aerobic performance
Source: BMC Evol Biol. 2015 Aug 26;15:173. doi: 10.1186/s12862-015-0453-7 (PMC4549853; doi:10.1186/s12862-015-0453-7)
Supplement: Additional file 1: Table S1. — Species list with mitochondrial genome accession number from NCBI. Table S2. PCR primers used for nuclear markers used in this study. Table S3. GenBank accession number of nuclear gene (rag1, rag2, tmo4c4, zic1, myh6, and btbd7) sequences of studied species. Figure S1. Phylogeny of six fish groups based on Bayesian and maximum likelihood methods. Figure S2. Summary of selection on all mitochondrial genes. Table S4. Number of amino acid sites showing positive selection signals in each mitochondrial gene on relevant branches. Table S5. Positively selected amino acid sites for each gene on various branches. (DOCX 351 kb) [file 12862_2015_453_MOESM1_ESM.docx]

**Supplementary Materials**

Figure S1 Phylogeny of six fish groups based on Bayesian and maximum likelihood methods. Maximum liklihood analyses were partitioned by codon position; Bayesian analyses were not partitioned. Values above each branch are from analyses of 13 mitochondrial protein genes. Values below each branch are from analyses of a subset of taxa for which 7 nuclear genes plus 13 mitochondrial genes were available. Bayesian posterior probabilities are listed on the left of the /with bootstrap percentages on the right.


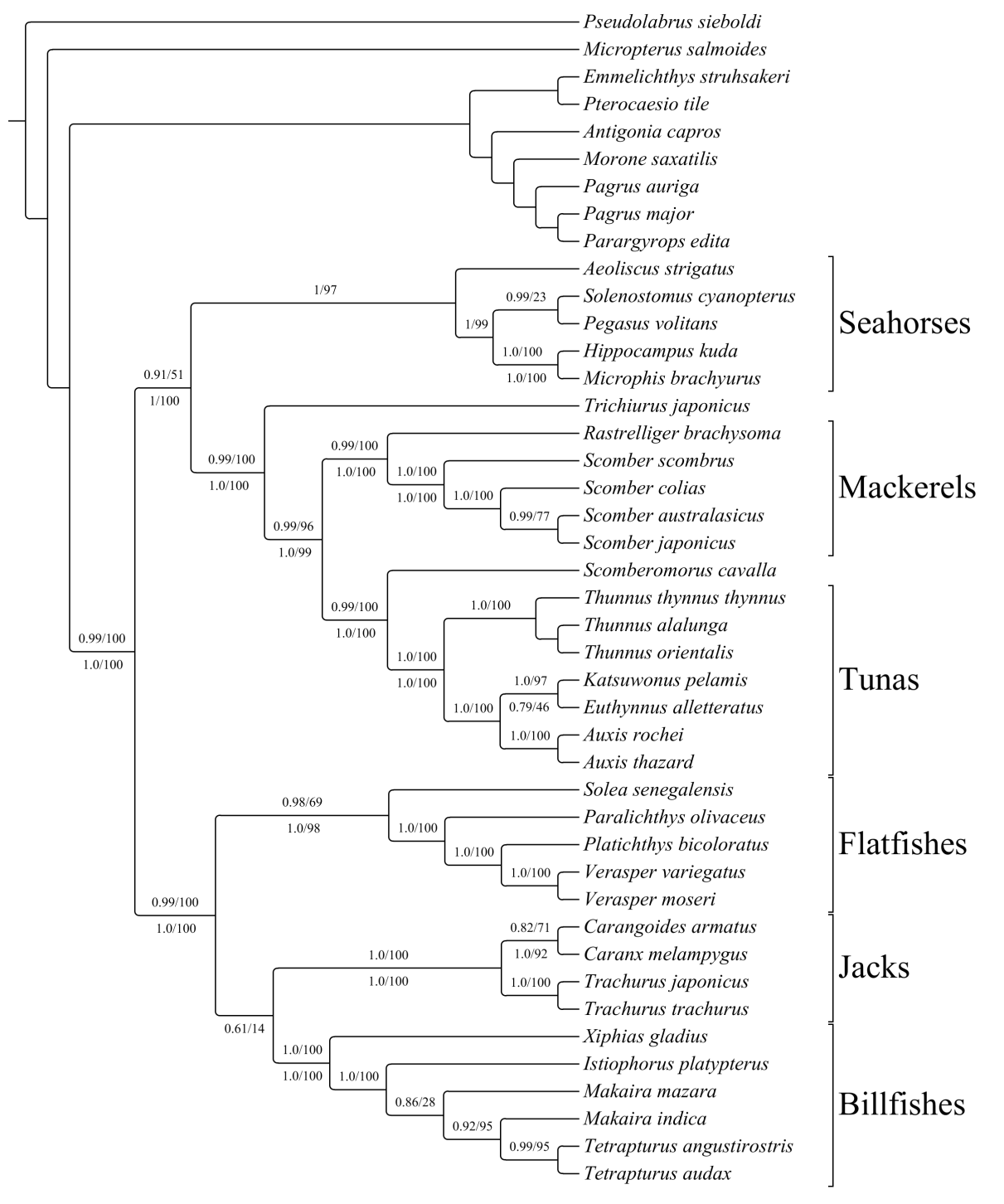


Figure S2 Summary of selection on all mitochondrial genes. Filled circles represent positively selected sites (p < 0.001) by TreeSAAP analysis. The dataset was analyzed for significance for the 31 physicochemical properties using a sliding window with size of 20 amino acid sites. The y-axis represents the number of properties for which that site was determined to be subject to positive natural selection.

ND6

ND4

ND3

COX2

ATP6

ND2

ATP8

CYTB

ND5

ND4L

COX3

ND1

COX1

Table S1 Species list with mitochondrial genome accession number from NCBI.

| Species | NCBI Accession |
| --- | --- |
| Aeoliscus_strigatus | NC_010270 |
| Antigonia_capros | NC_003191 |
| Auxis_rochei | NC_005313 |
| Auxis_thazard | NC_005318 |
| Carangoides_armatus | NC_004405 |
| Caranx_melampygus | NC_004406 |
| Emmelichthys_struhsakeri | NC_004407 |
| Euthynnus_alletteratus | NC_004530 |
| Hippocampus_kuda | NC_010272 |
| Istiophorus_platypterus | NC_012676 |
| Katsuwonus_pelamis | NC_005316 |
| Makaira_indica | NC_012675 |
| Makaira_mazara | NC_012680 |
| Microphis_brachyurus | NC_010273 |
| Micropterus_salmoides | NC_014686 |
| Morone_saxatilis | NC_014353 |
| Pagrus_auriga | NC_005146 |
| Pagrus_major | NC_003196 |
| Paralichthys_olivaceus | NC_002386 |
| Parargyrops_edita | NC_008616 |
| Pegasus_volitans | NC_010271 |
| Platichthys_bicoloratus | NC_003176.1 |
| Pseudolabrus_sieboldi | NC_009067 |
| Pterocaesio_tile | NC_004408 |
| Rastrelliger_brachysoma | NC_013485 |
| Scomber_australasicus | NC_013725 |
| Scomber_colias | NC_013724 |
| Scomber_japonicus | NC_013723 |
| Scomber_scombrus | NC_006398 |
| Scomberomorus_cavalla | NC_008109 |
| Solea_senegalensis | NC_008327 |
| Solenostomus_cyanopterus | NC_010267 |
| Tetrapturus_angustirostris | NC_012679 |
| Tetrapturus_audax | NC_012678 |
| Thunnus_alalunga | NC_005317 |
| Thunnus_orientalis | NC_008455 |
| Thunnus_thynnus_thynnus | NC_004901 |
| Trachurus_japonicus | NC_002813 |
| Trachurus_trachurus | NC_006818 |
| Trichiurus_japonicus | NC_011719 |
| Verasper_moseri | NC_008461 |
| Verasper_variegatus | NC_007939 |
| Xiphias_gladius | NC_012677 |

Table S2 PCR primers used for nuclear markers used in this study.

| Locus | Primer names | Primers (5’~3’) |
| --- | --- | --- |
| rag1 | rag1f1 | GAGCTTCTCCCHGGHTTTCA |
| rag1 | rag1r1 | AAGTGRAAGCGGAAGGAGCG |
| rag1 | rag1f2 | ATGAAAGAGAGCAGGCTYATC |
| rag1 | rag1r2 | AGGGCTGCCCTCCAGCTGCG |
| rag1 | rag1f3 | TCTSAAAACATGGTGCTDCA |
| rag1 | rag1r3 | CCATCTYTCTCKATGATTTC |
| rag2 | rag2f1 | TTCCAGAGAGYTAYCTCATC |
| rag2 | rag2r1 | AGCAARGGRCTGCCCTGCAG |
| rag2 | rag2f2 | GGACAGTCYTTCCATBTGGC |
| rag2 | rag2r2 | GGCARCATTTGATCCARTAGCC |
| tmo-4c4 | tmo4c4f1 | AAGAARAGAGTGTTTGAAAATG |
| tmo-4c4 | tmo4c4r1 | ACAGCWCCCTCCTCRTAAAT |
| tmo-4c4 | tmo4c4r2 | ATRATCATRCTCTTRTTGTC |
| zic1 | zic1f1 | ATGCTCTTGGACGCAGGACCGCA |
| zic1 | zic1r1 | CCACAGCGGGGAACGGACA |
| zic1 | zic1f2 | CATCACTCAACAGGCGAAG |
| zic1 | zic1r2 | TTGGCTTTGAACGGYTTYCCTTC |
| myh6 | myh6f1 | ATGCYTAYCARTACATGCTGAC |
| myh6 | myh6r1 | GGTTRATYCTVACCACCATCCA |
| myh6 | myh6f2 | GAGAACCARTCBGTSCTCATCAC |
| myh6 | myh6r2 | CCAGTTGAACATYTTYTCRTA |
| btbd7 | btbd7f145 | CCAGTCGCTCAGCTGATCATGC |
| btbd7 | btbd7r1093 | ATGTGGTANAGCTCCATNGCCTC |

Table S3 GenBank accession number of nuclear gene (rag1, rag2, tmo4c4, zic1, myh6, and btbd7) sequences of studied species.

|  | rag1 | rag2 | tmo4c4 | zic1 | myh6 | btbd7 |
| --- | --- | --- | --- | --- | --- | --- |
| Abudefduf_vaigiensis |  |  | KP866724 | KP866740 |  |  |
| Acanthocybium_solandri | KP866741 |  |  |  |  |  |
| Aphanopus_carbo | KP866742 | KP866761 | KP866710 |  |  |  |
| Auxis rochei | KP866743 | KP866762 | KP866703 |  | KP137552 |  |
| Brama brama |  | KP866763 | KP866725 | KP866734 | KP137555 |  |
| Carangoides_ferdau | KP866744 | KP866764 |  |  |  |  |
| Caranx_ruber |  | KP866765 | KP866704 |  |  |  |
| Centrolophus_sp. |  | KP866766 | KP866719 | KP866739 | KP137558 | KP121466 |
| Centropomus undecimalis |  |  |  | KP866730 | KP137554 |  |
| Centropomus_ensiferus |  |  | KP866709 | KP866731 |  |  |
| Elassoma_evergladei |  | KP866767 |  |  |  |  |
| Etheostoma_vitreum |  | KP866768 | KP866722 |  |  |  |
| Euthynnus_affinis | KP866745 |  |  |  |  | KP121462 |
| Gempylus_serpens | KP866746 | KP866769 | KP866712 |  |  |  |
| Gomphosus_varius |  |  | KP866721 |  |  |  |
| Gymnosarda_unicolor |  | KP866770 | KP866717 |  |  |  |
| Icichthys_lockingtoni | KP866747 | KP866771 | KP866718 | KP866738 | KP137557 | KP121465 |
| Katsuwonus_pelamis | KP866748 |  |  |  |  |  |
| Lepidopus_altifrons | KP866749 | KP866772 | KP866713 |  |  |  |
| Lycodes terraenovae |  | KP866773 | KP866708 |  | KP137553 |  |
| Micropterus_salmoides |  | KP866774 |  | KP866737 |  | KP121464 |
| Neoepinnula_americana | KP866750 |  | KP866711 |  |  |  |
| Paralichthys_californicus | KP866751 |  |  | KP866729 |  |  |
| Peprilus simillimus | KP866752 | KP866775 | KP866716 |  | KP137556 |  |
| Perca_flavescens |  | KP866776 | KP866723 |  |  |  |
| Platax_orbicularis |  |  | KP866720 |  | KP137559 |  |
| Pterycombus_brama |  | KP866777 | KP866715 | KP866735 |  |  |
| Ruvettus_pretiosus | KP866753 |  |  | KP866732 |  |  |
| Schedophilus_medusophagus | KP866754 | KP866778 |  |  |  |  |
| Scomber combrus |  |  |  | KP866726 |  | KP121459 |
| Scomber japonicus | KP866755 | KP866779 | KP866702 | KP866727 | KP137551 | KP121460 |
| Scomberomorus maculatus |  |  |  | KP866728 |  | KP121461 |
| Sphyraena_argentea |  |  | KP866707 |  |  |  |
| Sphyraena_barracuda | KP866756 |  |  |  |  | KP121463 |
| Sphyraena_putnamae | KP866757 |  | KP866706 |  |  |  |
| Taractichthys_longipinnis |  | KP866780 | KP866714 | KP866733 |  |  |
| Tetrapturus_albidus | KP866758 | KP866781 | KP866705 |  |  |  |
| Thunnus_albacares | KP866759 |  |  |  |  |  |
| Trachurus_lathami |  | KP866782 |  |  |  |  |
| Trichiurus_lepturus |  |  |  | KP866736 |  |  |
| Xiphias_gladius | KP866760 |  |  |  |  |  |

Table S4 Number of amino acid sites showing positive selection signals in each mitochondrial gene on relevant branches. The top number for each branch represents the number of amino acids having Bayes empirical Bayes (BEB) posterior probability higher than 0.95 identified in PAML; the bottom number represents the number of amino acids having BEB posterior probability higher than 0.80 and lower than 0.95 identified in PAML. The last row lists the total number of amino acid sites identified as positively selected by TreeSAAP. See supplementary table S4 for additional details.

| Branch | ND1 | ND2 | CO1 | CO2 | ATP8 | ATP6 | CO3 | ND3 | ND4L | ND4 | ND5 | ND6 | CYTB |
| --- | --- | --- | --- | --- | --- | --- | --- | --- | --- | --- | --- | --- | --- |
| b2 | 0 | 1 | 0 | 0 | 0 | 1 | 0 | 0 | 0 | 0 | 0 | 0 | 0 |
|  | 0 | 0 | 0 | 0 | 0 | 0 | 0 | 0 | 0 | 0 | 0 | 0 | 1 |
| b3 | 0 | 1 | 0 | 0 | 0 | 2 | 1 | 0 | 0 | 0 | 0 | 0 | 1 |
|  | 0 | 0 | 0 | 0 | 0 | 0 | 0 | 0 | 0 | 0 | 0 | 0 | 0 |
| b4 | 0 | 0 | 1 | 2 | 0 | 0 | 1 | 0 | 1 | 0 | 2 | 2 | 0 |
|  | 0 | 0 | 0 | 0 | 0 | 0 | 0 | 0 | 0 | 0 | 0 | 1 | 0 |
| b6 | 1 | 0 | 0 | 0 | 0 | 1 | 0 | 0 | 0 | 0 | 1 | 0 | 0 |
|  | 0 | 0 | 0 | 0 | 0 | 0 | 0 | 0 | 0 | 0 | 0 | 1 | 0 |
| b11 | 0 | 0 | 0 | 0 | 0 | 1 | 0 | 0 | 0 | 1 | 0 | 1 | 0 |
|  | 0 | 0 | 0 | 0 | 0 | 0 | 0 | 1 | 1 | 0 | 2 | 0 | 0 |
| b12 | 0 | 0 | 0 | 0 | 0 | 0 | 0 | 0 | 0 | 0 | 0 | 0 | 0 |
|  | 0 | 0 | 0 | 0 | 0 | 1 | 0 | 0 | 0 | 0 | 0 | 0 | 0 |
| b13 | 0 | 0 | 0 | 0 | 0 | 0 | 0 | 0 | 0 | 0 | 1 | 0 | 0 |
|  | 0 | 2 | 0 | 0 | 0 | 1 | 1 | 1 | 0 | 1 | 1 | 0 | 3 |
| b16 | 0 | 0 | 0 | 0 | 0 | 0 | 0 | 0 | 0 | 0 | 0 | 0 | 0 |
|  | 0 | 1 | 0 | 0 | 0 | 0 | 0 | 0 | 0 | 1 | 1 | 0 | 0 |
| b17 | 0 | 0 | 0 | 0 | 0 | 0 | 0 | 0 | 0 | 1 | 1 | 0 | 0 |
|  | 1 | 0 | 0 | 0 | 0 | 0 | 0 | 0 | 0 | 0 | 0 | 0 | 0 |
| b23 | 0 | 0 | 0 | 0 | 0 | 0 | 0 | 0 | 0 | 0 | 0 | 0 | 0 |
|  | 0 | 0 | 0 | 0 | 0 | 0 | 0 | 0 | 0 | 0 | 0 | 0 | 0 |
| b24 | 0 | 0 | 0 | 0 | 0 | 0 | 0 | 0 | 0 | 0 | 0 | 0 | 0 |
|  | 0 | 0 | 0 | 0 | 0 | 0 | 0 | 0 | 0 | 0 | 0 | 0 | 0 |
| b25 | 1  0 | 2  1 | 0  0 | 0  0 | 0  1 | 0  0 | 0  0 | 0  0 | 0  1 | 2  2 | 1  1 | 0  2 | 0  0 |
| Total in  PAML (>0.95) | 2 | 4 | 1 | 2 | 0 | 5 | 2 | 0 | 1 | 4 | 6 | 3 | 1 |
| Total in  PAML (<0.95 >0.8) | 1 | 4 | 0 | 0 | 1 | 2 | 1 | 2 | 2 | 4 | 5 | 4 | 4 |
| Total in TreeSAAP | 183 | 77 | 25 | 32 | 26 | 44 | 24 | 20 | 11 | 82 | 152 | 46 | 42 |

Table S5 Positively selected amino acid sites for each gene on various branches. Each site lists the ordinal number of this site in the aligned sequence matrix, and the amino acid (based on the top sequence in the alignment). Symbols indicate bayes empirical bayes (BEB) probabilities as follows: ** >0.99, * 0.95 - 0.98, # 0.80 - 0.94, no symbol 0.5 - 0.79. If the site is also identified as having at least one property significant in TreeSAAP, the number of such properties is shown in square brackets. Sites of each gene in the same color indicate they occur on multiple branches.

|  | ND1 | ND2 | COI | COII | ATP8 | ATP6 | COIII | ND3 | ND4L | ND4 | ND5 | ND6 | CYTB |
| --- | --- | --- | --- | --- | --- | --- | --- | --- | --- | --- | --- | --- | --- |
| b2 | 10 I | 419 L [9]  475 Q  602 L^*^ [2] | 1074 T | 1343 V |  | 1596 N^*^ [6] |  | 2007 S |  | 2306 I  2363 S  2404 V [3]  2423 I  2472 A  2525 I | 3172 I [3] |  | 3627 A^#^ |
| b3 | 3 S  29 V [2]  159 I [3]  177 T  274 L | 371 N  595 T  652 I  654 S^*^ | 847 P  1005 I  1185 Q |  | 1441 T [2]  1466 S [6] | 1520 G [7]  1524 M^*^[7]  1527 T  1529 Q  1531 L  1534 L  1536 L  1596 N^*^ [6]  1652 F [10] | 1854 E^*^  1857 Q |  | 2111 G  2122 L | 2224 C [2]  2556 I [2] | 2643 S [2]  3078 P  3176 L | 3330 W  3346 A  3347 L [1]  3364 E [1]  3365 V  3415 L | 3475 S  3477 I [2]  3478 A  3479 T  3482 S  3500 M  3576 N [9]  3580 Q  3613 L [2]  3632 D  3652 G^*^[1]  3655 T  3680 L  3724 I  3739 T [1]  3761 V  3763 P  3784 T [5] |
| b4 | 181 I | 435 L [2]  517 V [3]  559 T [1]  563 A [1]  602 L [2]  641 L [4]  658 I | 849 I  851Q^**^ [4]  854 T  1187 Q | 1205 V  1236 S  1237 T  1241 N  1243L^**^[3]  1333 V  1361 M  1372 T  1375 T  1376L^**^[8]  1416 L [4] |  | 1550 I | 1706 A  1750 L [3]  1760 I  1791 F  1870 F^*^ [9]  1924 Y |  | 2100 T  2205 F | 2278 A [7]  2530 A  2611 I | 2656 I  2697 S [5]  3214 I [3]  3220 A [4]  3239 V^**^[14] | 3248 Y  3251 I  3253 L  3256 S^**^  3390 V | 3460 I[6]  3482 S  3627 A[2] |
| b6 | 7 T^#^ [7]  259 E [9] |  |  | 1238 K  1244 L |  | 1483S^*^ | 1746 I [6]  1922 I |  |  |  | 2635 H^*^[8] | 3249 V [1] |  |
| b11 |  |  |  |  |  | 1483S^*^ |  | 2050 F^#^ [2] | 2080 V^#^ [9] | 2192 P^*^ [3] | 2710 L^#^ [3]  2722^#^ I [11] | 3336 M^*^[3] |  |
| b12 |  |  | 891 T  1133 I [1] | 1239 L | 1433 L [1] | 1503 F [2] | 1740 I [1] |  | 2100 T | 2612 A [6] |  |  | 3421 S [8] |
| b13 | 9 I | 332^#^ I [1]  601^#^ M |  |  |  | 1483^#^ S  1543 L [3] | 1740 I [1]  1746 I [6]  1750 L [3]  1754 Y [2] | 2054 S [3] |  | 2228 K^#^[11] | 2656 I  2661 S [2]  3070 S^#^ [2]  3152 F^*^ [9]  3240 L [1] |  | 3543 A [9]  3611 T^#^ [3]  3714 S^#^ [10]  3794 M [4] |
| b16 |  | 411 D^#^ [2] |  |  | 1451 K |  |  |  |  | 2568 L^#^ | 2690 F [8]  2846 M^#^ |  | 3627 A [2]  3714 S [10] |
| b17 | 177 T^#^ | 411 D [2]  470 P [2] | 935 A [4] | 1289 L |  |  |  | 1980 F |  | 2564 P^*^ | 2915 L^*^ [7] |  |  |
| b23 |  | 475 Q  633 I [6] |  |  | 1467 K [12] | 1544 M |  |  |  | 2473 A [2] | 2657 I [1]  2910 P [5]  3208 T [0] |  |  |
| b24 |  | 398 S |  |  |  | 1500 W |  | 2042 L | 2124 T  2167 Q | 2318 N | 2702 T [7]  2717 S [6]  3214 I [3] |  | 3464 L  3609 A |
| b25 | 158 A^*^ [7] | 417 T [2]  585 G^*^ [6]  592 S^*^ [10]  600 N^#^ [2]  656 M [11]  670 T [1] |  |  | 1440 A^#^ [7]  1459 V [2] | 1663 L |  |  | 2168 A^#^ [6] | 2217 W^**^  2305 L  2364 T^#^  2572 L^*^ [2]  2633 F^#^ [10] | 2654 T^#^ [6]  2823 S [16]  3148 S^*^ [2]  3153 T [1]  3161 L [13] | 3291 F^#^ [2]  3330 W  3347 L [1]  3370 L [9]  3411 E^#^ [19] | 3543 A [9] |
